# Children and Pet Animals

**Published:** 1887-09

**Authors:** T. W. Higginson


### CHILDREN AND PET ANIMALS.

A child brought up without the knowledge of pet animals is a solitary being, no matter if there be brothers and sisters, while a child who has animals to tend is never quite alone. A dog is of itself a liberal education, with its example of fidelity, unwearied activity, cheerful sympathy and love stronger than death ; nay, love that is triumphant over shame and ignominy and sin—influences that so often wear out human love or make it change to hate. How many of us hold to our friends with a love as inexhaustible and unextinguishable as that which our dog gives to us ? The child especially finds in the faithful creature much of its own impulsive and ardent life ; the delight in little things, the ready curiosity, the ceaseless activity, the quick changes of occupation, the unabated interest in existence. Kittens, again, seem sent to give to a child just what the dog leaves out ; the more refined ways, the soft playfulness, the gentle domesticity, the willingness to be tended and petted. Kittens about the house supply the smaller punctuation in the book of life ; their little frisks and leaps and pats are the commas and semi colons and dashes, while the big dog puts in the colons and periods.

Animals, again, give to us, even by what they receive and evoke from us, the habit of care and tenderness. Those petted dogs we see carried in the arms of young girls in fashionable equipages, are rarely a substitute for the natural object of such emotion, they are rather a preparation or intermediate possession that precedes it ; something that is more than a doll and less than a human child. Mr. Carnegie tells us that he saw at a large New York stable, a card nailed up giving for the coachman the address of the proper physician to be called upon, if the favorite dog should be ill. He also tells us of a young lady who, having to go on a journey, had to leave her favorite collie to some one's special attention, and Mr. Carnegie suggested that, as he had given her the dog, it might be perfectly safe to leave her with him, "or rather, with Jack and the horse." With a grave shake of the head, she answered, "I have thought of that ; but it won't do ; he requires a woman's care." Here the woman and the favorite met on equal terms ; neither could do without the other. The care given by the young girl was simply the anticipated tenderness of a mother for a child.

The self-control that must be learned in dealing with animals, is in itself an education. One of the child's first lessons in governing its impulses is when it finds that the kitten cannot be caught by running and shouting, but by quiet and measured approaches. The control of animals, from the lamb to the lion, is not a matter of force, but of gentleness and a steady eye. Impulses that seem the very strongest in animals, as the disposition of dogs to chase cats or bite, can be better overcome by accustoming them very early to the sight and touch of the weaker creatures than by any blows. All this is a lesson to the child, and it unconsciously learns the application to itself. In days when oxen were employed largely on our farms it used to be a common thing at a "cattle show," to see some sunburnt farmer's boy drive in a yoke of half-grown steers, and win the admiration of all the men by the gentle skill with which he handled them. On a farm near my summer home there is a fine bull, which is better controlled and led by a boy of thirteen than by anybody else. There surely is, as Heine says, an occult sympathy between children and animals, as between two races not sundered very long ago.—*T. W. Higginson, in Harper's Bazar.*

